# Study protocol of physical activity and sedentary behaviour measurement among schoolchildren by accelerometry - Cross-sectional survey as part of the ENERGY-project

**DOI:** 10.1186/1471-2458-11-182

**Published:** 2011-03-25

**Authors:** Mine Yıldırım, Maïté Verloigne, Ilse de Bourdeaudhuij, Odysseas Androutsos, Yannis Manios, Regina Felső, Éva Kovács, Alain Doessegger, Bettina Bringolf-Isler, Saskia J te Velde, Johannes Brug, Mai JM Chinapaw

**Affiliations:** 1 Department of Public and Occupational Health and the EMGO Institute for Health & Care Research, VU University Medical Center, Amsterdam, the Netherlands; 2 Department of Movement and Sport Sciences, Ghent University, Ghent, Belgium; 3 Department of Nutrition and Dietetics, Harokopio University, Athens, Greece; 4 Department of Paediatrics, University of Pécs, Pécs, Hungary; 5 The Federal Institute of Sport, Magglingen, Switzerland; 6 Department of Epidemiology and Public Health, Swiss TPH and University of Basel, Switzerland; 7 Department of Epidemiology and Biostatistics and the EMGO Institute for Health & Care Research, VU University Medical Center, Amsterdam, the Netherlands

## Abstract

**Background:**

Physical activity and sedentary behaviour among children should be measured accurately in order to investigate their relationship with health. Accelerometry provides objective and accurate measurement of body movement, which can be converted to meaningful behavioural outcomes. The aim of this study was to evaluate the best evidence for the decisions on data collection and data processing with accelerometers among children resulting in a standardized protocol for use in the participating countries.

**Methods/Design:**

This cross-sectional accelerometer study was conducted as part of the European ENERGY-project that aimed to produce an obesity prevention intervention among schoolchildren. Five countries, namely Belgium, Greece, Hungary, Switzerland and the Netherlands participated in the accelerometer study. We used three different Actigraph models-Actitrainers (triaxial), GT3Xs and GT1Ms. Children wore the device for six consecutive days including two weekend days. We selected an epoch length of 15 seconds. Accelerometers were placed at children's waist at the right side of the body in an elastic belt.

In total, 1082 children participated in the study (mean age = 11.7 ± 0.75 y, 51% girls). Non-wearing time was calculated as periods of more than 20 minutes of consecutive zero counts. The minimum daily wearing time was set to 10 hours for weekdays and 8 hours for weekend days. The inclusion criterion for further analysis was having at least three valid weekdays and one valid weekend day. We selected a cut-point (count per minute (cpm)) of <100 cpm for sedentary behaviour, <3000 cpm for light, <5200 cpm for moderate, and >5200 cpm for vigorous physical activity. We also created time filters for school-time during data cleaning in order to explore school-time physical activity and sedentary behaviour patterns in particular.

**Discussion:**

This paper describes the decisions for data collection and processing. Use of standardized protocols would ease future use of accelerometry and the comparability of results between studies.

## Background

The accurate measurement of physical activity (PA) and sedentary behaviour is essential to evaluate their health impact in terms of amount and intensity [1,2]. The complex nature of PA that covers both sports and non-sport activities makes it complicated to perfectly measure all of its aspects. Ideal measurement should be applied in daily life, lasting long enough to represent habitual activity patterns and with low inconvenience to the participant [3]. Self-reported measures may not accurately reflect free-living activity patterns due to recall bias and/or its susceptibility to reporting bias by social desirability [4,5]. Furthermore, a recent systematic review reported that there is currently no PA questionnaire for youth with both acceptable reliability and validity [6].

Apart from the importance of PA to maintain good health, there is growing evidence that excessive sedentary behaviour is linked to a range of health problems in particular overweight and obesity [7-9]. Sedentary behaviours such as sitting and screen-based entertainment include activities that do not increase energy expenditure substantially above resting level [10]. Since sedentary behaviour such as TV viewing has a strong habitual element (a kind of automatic behaviour that is done without thinking) it is necessary to measure it over a period of time [11]. It has been shown that using single-item self-reports or selecting only television viewing as an indicator of sedentary behaviour does not estimate the broad range of sedentary behaviours in which children participate [12,13].

Over the last decades, motion sensors have been widely used as objective measures of PA as well as sedentary behaviours to overcome the problems related to self-report methods [14]. The recent technological advances increase the capabilities of motion sensors such as accelerometers, which are small and light devices that are able to collect data for weeks [3]. Accelerometers measure accelerations caused by body movements in one to three orthogonal planes (vertical, mediolateral and anteroposterior) and basically generate the activity counts and minutes spent above predefined thresholds [15]. Using accelerometers allows researchers to focus on activity patterns rather than energy expenditure [16]. In addition, they provide data to further investigate the dose-response relationship between activity patterns and health and providing evidence to be used in public health recommendations [17].

Recent reviews reported that accelerometers provide an accurate and precise measurement of all intensity levels of PA with the entire range from sedentariness to very vigorous [11,15,18]. Distinguishing light activity and sedentary behaviour is important due to the contribution of total daily light activity to energy expenditure. Accelerometers can make this differentiation that eases to study the relationship between sedentary behaviour and health [10]. A central issue in using accelerometers is careful planning of data collection, data analysis and interpretation [15]. Unfortunately to date there is no consensus on the data analysis and interpretation of accelerometers. In 2004, an international conference was held to close the knowledge gap in the science of accelerometry. As a result, the best practice recommendations were derived on accelerometer use but still there was no clear consensus [19]. For this reason, unresolved issues in methodology and data processing should be taken into account in the research planning phase. Therefore, the current paper aims to critically appraise the best evidence for the decisions on data collection and data processing with accelerometers among children. With this protocol we also aimed to standardize the implementation of the accelerometer study between participating countries.

In the "EuropeaN Energy balance Research to prevent excessive weight Gain among Youth" (ENERGY)-project, we objectively assessed PA and sedentary time by accelerometers in a subsample of about 1000 children aged 10-12 from five European countries. This paper describes the protocol of the data collection, processing and analysis.

## Methods

### Project overview

The ENERGY-project is a cross-European project aimed at the systematic development of a cross-European school-based and family involved intervention program to prevent overweight among children. The project is carried out by a multidisciplinary team of researchers from eleven European countries and Australia. The design and conceptual framework of the ENERGY-project have been previously described [20]. As part of the development of this obesity prevention program, cross-sectional school-based surveys were conducted among 10-12 year old children and their parents in eight European countries (Belgium, Greece, Norway, Hungary, Slovenia, Spain, Switzerland and the Netherlands). The aim of the survey was to provide up to date information on the prevalence of overweight and obesity - measured by objective measures of height, weight and waist circumference-, on the most important energy balance related behaviours (EBRBs) and their social, cognitive and school environmental determinants. Schools were employed in each country to reach 1000 eligible children. The schools were randomly selected concerning the degree of urbanization of the different provinces and the socioeconomic status (SES) of the different areas within the selected provinces. All participating countries obtained ethical committee approvals. The details on the survey are described elsewhere [21].

### Accelerometry study

In five countries - Belgium, Greece, Hungary, Switzerland and the Netherlands - accelerometer data were collected in a subsample of approximately 200 children per country. The selection of schools was balanced across the participating provinces selected for the main survey. The distribution of the number of selected schools for accelerometry was proportional to the number of schools in the selected cities (thus more schools from larger cities). One identical recruitment letter was sent to all randomly selected schools explaining that in some schools children would be asked to wear an accelerometer during six consecutive days. The selection of the schools for accelerometer data collection was based on the logistic planning of the data collection process as well as willingness of the schools. Trained researchers collected accelerometer data according to a standardised protocol.

### Type of accelerometers

We used three different models of Actigraph (Pensacola, FL) accelerometers namely Actitrainers (triaxial), GT3Xs and GT1Ms. The GT3X and GT1M (dimensions: 3.8 cm × 3.7 cm × 1.8 cm) and Actitrainer (dimensions: 8.6 cm × 3.3 cm × 1.5 cm) are lightweight devices weighing 27, 27 and 51 grams, respectively [22]. A recent study confirmed that vertical axis output of GT1M and GT3X was similar [23]. Actigraph accelerometers have shown adequate reproducibility, validity and feasibility in children and adolescents [18].

### Epoch length and duration of measurement

The accelerometer collects and sums the activity counts over a user-defined epoch [24]. Bailey et. al. [25] reported that children tend to have very short bursts of high intensity activities. In their study sample, the median duration of a high intensity activity was 3 seconds with 95% lasting <15 seconds. Therefore, to be able to accurately capture the pattern of high intensity and short duration PA, we selected a time interval/epoch length of 15 seconds.

The duration of the measurement should reflect habitual activity and variability in activity and sedentary behaviour patterns from day to day [15]. To acquire a valid representation of activity for children and adolescents four to nine days of monitoring are needed. Due to differences between weekday and weekend activity patterns, it is advised to combine weekdays and weekend days in measurement protocols [24]. Based on these findings, we collected data during six consecutive days including two weekend days.

### Initialising the devices

The ActiLife software (at least version 4.3.0) was used to initialise the accelerometers and upload the collected data [26]. Before initialising, the devices were fully charged by connecting the device to a standard USB port. The start date was set at the day when the devices were handed out to the children starting at 05.00 AM. We did not select a stop time. For optional modes dual axis and third axis were selected for GT3X and Actitrainers additional to activity mode.

### Placement of devices

Godfrey et. al. [27] emphasized the necessity of studying 'whole body' movements presented by placement of a motion sensor as close as possible to the centre of mass of the body, such as the waist. Children were fitted with accelerometers located at the waist at the right side of the body in an elastic belt. Children were asked to wear the device either under or on their clothes during all waking hours, apart from water-based activities. We marked the top of the device with a sticker and instructed the children to be aware that the accelerometer should be placed with the sticker on top.

### Diaries (log-books)

We asked children to fill in a diary recording the time of getting up in the morning and going to bed for sleeping. They also noted the time and reason why the device was removed for 5 minutes or more for any activity such as swimming, or showering. We also asked whether the measurement day was a school day, half-school day or non-school day (See additional file 1: Diary).

### Pre-testing the protocol

The researchers who were responsible for the accelerometer data collection were trained to work according to a standard protocol. The participating countries pre-tested the protocol with at least five 10-12 year old children for feasibility. Based on the pre-test results, some changes were made in the layout of the diary. We emphasized that children need to fill in the diaries themselves and that the devices should also be worn during all activities except water activities.

### Data collection

Researchers distributed the accelerometers face-to-face at schools. Information about accelerometer use was given to the children orally and at the end of the information session accelerometers were handed out. Researchers placed the accelerometer to the children's waist. Additionally, children and parents received a brochure about accelerometer use including the instructions for children (See additional file 2: instructions and information brochure) and the diary. Teachers were also informed about the procedure and asked to remind the children to wear the devices every day. Researchers called and asked the teachers one day before collecting the devices to remind children to bring the device and diary back to school. After the proposed wearing period, children brought the device back to school and handed it out to their teacher. Downloading the data from accelerometers was done as soon as possible on the same computer where it was initialised to prevent disturbances that can be caused by the time offset between computers. The diary data were entered manually in excel files.

Table 1 shows the characteristics of the children who participated in the accelerometer study per country. Sixty-seven percent of children (n = 722) had enough data to be included in further analyses.

**Table 1:** Characteristics of children who participated in the accelerometer study per country

Country	Initial number	Final number*	Age^a ^ (mean ± SD)	BMI^a ^ (mean ± SD)	Gender^a ^ (girl%)
Belgium	196	107	11.4 (0.7)	18.0 (2.6)	55.7
Greece	215	160	11.3 (0.6)	20.9 (3.9)	53.8
Hungary	194	147	12.2 (0.7)	19.9 (3.3)	50.0
Switzerland	277	206	11.4 (0.9)	17.8 (2.8)	58.0
the Netherlands	200	102	11.8 (0.6)	18.6 (3.0)	43.4

**TOTAL**	1082	722	11.7 (0.7)	18.9 (3.4)	53.2

* The number of children after data reduction

^a^Based on the final number of children

## Data Processing

All data collected in the study were transferred to VU University Medical Center (VUmc), the Netherlands for data processing. Meterplus [28] was used for data reduction and analysis. Meterplus is a Windows-based program developed by researchers from San Diego State University in partnership with Actigraph. The Meterplus program was used to prepare and clean the accelerometer data files according to non-wearing time (as described below), invalid data (i.e. days that have not enough wearing time and implausibly high count values) and specific activity bout definitions such as bouts of sedentary time.

### Data reduction

It is known that PA and sedentary time results may change substantially depending on how data is processed [29, Fischer et. al. unpublished data]. Therefore, the quality of the accelerometer data should be checked first using systematic data reduction procedures. The decisions on minimum daily wearing time and number of required days for data analysis are critical data reduction issues. Wearing time calculation is not solely excluding all zero count values from data, since sedentary behaviour is part of data. For this reason decisions should be made regarding duration of consecutive zeros to distinguish between non-wearing and sedentary time [30]. Non-wearing time is the time period that participants did not wear the device such as during sleeping or water sports/activities. In the current study non-wearing time was calculated as periods of more than 20 minutes of consecutive zero counts [31,32]. Wearing time was calculated by subtracting non-wearing time from 24 hours [33]. The minimum daily wearing time was set at 10 hours for weekdays and 8 hours for weekend days considering different sleep patterns at weekends.

Another critical issue is to include enough days to be able to reflect children's habitual PA pattern. A valid day requires a minimum number of hours of wearing. Children who had at least three valid weekdays and one valid weekend day were included in the further data analysis [34].

### Cut-points for activity categories

The end results from accelerometer measurement are count values. Counts have been calibrated against energy expenditure in order to get a biological meaning [15]. The most common way to estimate time spent in a specific activity category is to develop a regression equation that defines the relationship between counts and energy expenditure, allowing counts to be converted to units of energy expenditure. To date, a number of calibration equations for youth were developed for the Actigraph accelerometers, though the methods used were rather different [35]. Table 2 shows the most common used cut-points that are appropriate for children aged 10-12.

**Table 2:** Actigraph accelerometer cut-points that are appropriate for children aged 10-12

Study	Cut-points (counts/minute)				
Authors (year)	Sample	Sedentary	Light	Moderate	Vigorous
Freedson et. al.* (2005)	6-18 y, 80 children	-	100-2219	2220-4135	≥4136
Puyau et. al. (2002)	6-16 y, 26 children	≤800	801-3199	3200-8199	≥8200
Treuth et. al. (2004)	13-14 y, 74 girls	≤100	101-2999	3000-5199	≥5200
Mattocks et. al. (2007)	12 y, 163 children	-	-	3581-6129	≥6130

* Age specific equation, the cut-points for 12 years of age.

Cut-points by Freedson et. al. [36] are completely laboratory-based. Puyau et. al. [37] and Treuth et. al. [38] used more free living activities, ranging in intensity from sedentary to vigorous. Mattocks et. al. [39] also used free living activities but only for moderate and vigorous activity intensities. Treuth et. al. [38] measured energy expenditure at rest and during activity with portable indirect calorimetry and used resting energy expenditure to establish MET-levels. This is important since children have higher resting metabolic rates than adults. A recent study comparing the different cut-points for sedentary behaviour (100, 300, 800, 1100) with the observation of children's behaviour showed that Actigraph cut-point of ≤100 cpm is the most appropriate one for quantifying time children spent on sedentary activities (Fischer et. al. unpublished data). Another study by Trost et. al. [40] evaluated the classification accuracy of cut-points (including those showed in Table 2) using energy expenditure measured by indirect calorimetry. They reported that the cut-points from Freedson et. al. [36] and Treuth et. al. [38] showed good classification accuracy. They also reported that 100 cpm as a cut-point for sedentary behaviour showed good to excellent classification accuracy. Therefore, we selected the cut-points from Treuth et. al. [38] for sedentary and activity classifications.

We also set a cut-point for the upper limit of count values to avoid spurious data based on the recommendations from Esliger et. al. [16]. It was shown that count values higher than 15.000 per minute are very unusual and implausible. For this reason the count values higher than 15.000 per minute were considered as missing values.

### Bouts of activity

Bouts of activity are sustained periods of elevated counts. Bouts of sedentary time are sustained periods of low counts. MeterPlus provides information on bouts of a specific intensity such as the frequency, when the bouts occurred, and how long they lasted. Analysis of bouts provides information on possible health benefits of the accumulation of certain intensity bouts [16]. We selected to analyse sedentary behaviour bouts lasting at least ten continuous minutes with two minutes tolerance.

### Outcome measures

We calculated total counts per day (the volume of activity), mean counts per minute (total counts divided by wearing time), and the amount of time spent in sedentary, and in light, moderate and vigorous intensity PA based on the vertical axis counts. We calculated the vector magnitude, a composite movement score for all three directions ([x^2 ^+y^2 ^+z^2^] ^0.5^).

### Time filters

We did not create time filters for sleeping time because this (as there are ≥20 minutes of consecutive zeros) is automatically coded as "non-wearing" time in Meterplus. We did not impute missing time because the review of Esliger et. al. [16] concluded that in many cases duration of the non-wearing time obtained by diaries is ambiguous. In addition, imputation conflicts with the objectivity of accelerometer data.

#### *School-time time filters*

Schoolchildren spend 57% of their waking time at school and school time PA opportunities accounts for >70% of moderate and vigorous PA/day for children [41]. Therefore, we plan to analyse school-time activity and sedentary behaviour patterns apart from the total day. For this reason, we created time filters for school-times during data cleaning in Meterplus. We collected the exact school-times from the school management forms filled out by the manager of each school. We used the same data processing criterion of a recent study from Van Sluis et. al. [42] and children who did not reach 500 minutes collected on at least 2 weekdays were excluded from the school-time analysis. We obtained the total valid hours per school day and the total time spent in sedentary behaviour, light, moderate and vigorous activity. We calculated the mean percentages of time spent in sedentary, moderate and vigorous activity categories per school day.

## Discussion

The cross-European survey in the ENERGY-project aimed to identify the potential determinants of EBRBs and the differences between countries regarding these determinants among schoolchildren and their parents. The accelerometer sub-study provides an objective measurement of PA and sedentary behaviour of children. With the systematic development of the accelerometer study protocol, we aimed to use the best evidence for the decisions on data collection and data processing with accelerometers. We also standardised the use of accelerometer**s **between the participating countries including data collection and data analysis.

To examine health impacts of PA and sedentary behaviour, researchers transfer accelerometry count values to behavioural outcome such as time spent in a specific activity category. It is important to select appropriate data processing criteria. For example, applying different cut-points to the same data significantly changes the estimation of activity and sedentary time [43,44]. This will affect the relationship of activity and sedentary time with a specific health outcome. To choose an evidence based cut-point, we summarised and evaluated existing paediatric calibration studies. When selecting cut-points for the activity categories the age group of study sample needs to be taken into consideration as the same cut-point may not work well across different age groups [10]. In the current study we have only one age group, i.e. 10-12 years old.

Furthermore, a recent study that used NHANES 2005-2006 accelerometry data showed that applying stricter compliance criteria during data processing resulted in somewhat higher moderate to vigorous PA levels [29]. In the current study we had to exclude nearly 30% of children from further analyses due to insufficient valid days. More studies on differences between different compliance criteria are needed, for example, the effect of lowering the minimum of three weekdays to two weekdays.

Another critical issue with accelerometer use is compliance to the protocol. Sirard and Slater [45] compared different methods among high school students to increase compliance such as giving incentives, diary and telephone calls. They showed that giving a diary to note down non-wearing time and reasons is an effective, low cost method to increase compliance. We used the diary method resulting in 67% compliance, which is worse to what has been found before by Sirard and Slater [45]. It indicates the need for additional compliance strategies for younger children.

One of the strengths of the accelerometer study is a large sample size from different countries in Europe that provides an overview of activity patterns across countries. Researchers should be aware of weaknesses of accelerometry use as well. Some of the limitations of accelerometers are not measuring the movement of arms in terms of carrying, lifting weights, not distinguishing between sitting, lying, and standing still and poor measurement of cycling behaviour. Furthermore, water-based activities cannot be measured by accelerometry. For the selection of accelerometer type several issues should be considered such as costs, software support, comparability with other studies and device features [46]. In the current study we selected the Actigraph models due to their common use and good psychometric properties against other accelerometer types among children [18].

To our knowledge, there is no validation study of vector magnitude value for children. Since it contains information of body movements of all three planes, it may increase the accuracy of the behavioural outcomes. There is a need for future studies on validation of vector magnitude for children. Even though there is best practice evidence on accelerometry use, there is still no consensus on several issues. For example, the number of days that are needed for school children to reflect their habitual activity patterns is not clear. Another critical issue that needs further study is missing data imputation using diary data. We decided not to impute data due to the ambiguity of diary data and also unknown mechanisms of missingness of the accelerometer data. Catellier et. al. [47] indicated that imputation of missing data would bias the result when missingness is not random, such as people less likely to wear the device when they are more inactive. Future studies might explore the usefulness of data imputation and comparison of different techniques of imputation with accelerometer data such as a recent study from Ottevaere et. al. [48].

## Conclusion

This evidence-based protocol of accelerometry use in children may guide future studies in the field of PA and sedentary behaviour measurement. The decisions on data collection and processing should be sample-specific and based on critical evaluation of available evidence. Despite its shortcomings, the accelerometry measurement is an advantageous method in PA and sedentary behaviour measurement among children. Further study on the unresolved methodological issues such as validation of vector magnitude and imputation techniques is recommended.

## Competing interests

The authors declare that they have no competing interests.

## Authors' contributions

MJMC was the principal investigator of the accelerometer study and designed the implementation. YM coordinated the cross-European survey in the ENERGY-project. MY, MV, OA, RF, AD and BBI took part in the data collection. MY and MV worked on the data processing of the accelerometer data. This protocol paper was written by MY, with input of all co-authors. All authors read and approved the final manuscript.

## Pre-publication history

The pre-publication history for this paper can be accessed here:

http://www.biomedcentral.com/1471-2458/11/182/prepub

## Supplementary Material

Additional file 1 **Diary**. Click here for file

Additional file 2 **Instructions and information brochure**. Click here for file
