# Supplementary material for: Study protocol of physical activity and sedentary behaviour measurement among schoolchildren by accelerometry - Cross-sectional survey as part of the ENERGY-project
Source: BMC Public Health. 2011 Mar 25;11:182. doi: 10.1186/1471-2458-11-182 (PMC3078096; doi:10.1186/1471-2458-11-182)
Supplement: Additional file 1 — Diary. [file 1471-2458-11-182-S1.PDF]

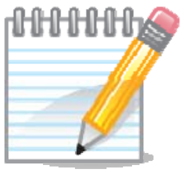

Institute Name and Contact Information

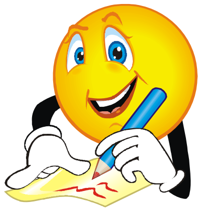

KIDS-METER LOG

At the next table, you see an example of how to fill in the diary for the kids meter. Write down the dates and days on which you wear the kids-meter. Note the times that you put it on and take it off during each day for getting up in the morning and going to bed for sleeping. If you put it off for more than 5 minutes please write this down below and for what reason (e.g. swimming, etc.)

Sample Entry

Day	Tuesday	
Date	March 1, 2010	
School Day	Yes (x) No ( ) Half-day ( )	
	Get up	Sleeping
Time	7.30	21.00
Activity	Start time	End time
1.Swimming	10.00	10.40
2.		

DON'T FORGET TO FILL THIS TABLE IN DURING THE NEXT 6 DAYS

Day												
Date												
School Day	Yes ( ) No ( ) Half-day ( )		Yes ( ) No ( ) Half-day ( )		Yes ( ) No ( ) Half-day ( )		Yes ( ) No ( ) Half-day ( )		Yes ( ) No ( ) Half-day ( )		Yes ( ) No ( ) Half-day ( )	
	Get up	Sleeping	Get up	Sleeping	Get up	Sleeping	Get up	Sleeping	Get up	Sleeping	Get up	Sleeping
Time												
Activity	Start time	End time	Start time	End time	Start time	End time	Start time	End time	Start time	End time	Start time	End time
1.												
2.												
3.												

RESEARCHER WILL FILL IN THIS PART

Country Code:
School Code:
Class Code:
Date of Birth (Age):

Student Code:
Serial number:
Date of distributing:
Date of collecting back:
