# Supplementary material for: Study protocol of physical activity and sedentary behaviour measurement among schoolchildren by accelerometry - Cross-sectional survey as part of the ENERGY-project
Source: BMC Public Health. 2011 Mar 25;11:182. doi: 10.1186/1471-2458-11-182 (PMC3078096; doi:10.1186/1471-2458-11-182)
Supplement: Additional file 2 — Instructions and information brochure. [file 1471-2458-11-182-S2.PDF]

KIDS-METER INFORMATION LETTER FOR PARENTS

Dear Parents,

Thank you for participating in the ENERGY project. As part of the ENERGY project your child will wear a kids-meter during 6 days. The purpose of this letter is to inform you how your child should wear this kids-meter.

What is a Kids-Meter?
The Kids-Meter measures all the movements of your child. It is a mechanical device without radiation. We are interested in what kind of movements children like to do.

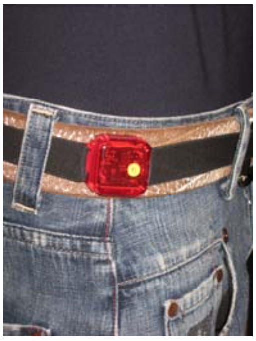

How to wear the Kids-Meter?
The Kids-Meter (attached to a belt) must be worn on the right waist; directly on the hip bone. Your child can wear it under or on his/her clothes. Please, make sure that the belt is tight enough (not too loose, and not too tight).

How long and When to wear the Kids-Meter?
Please remind your child to wear the Kids-meter for 6 consecutive days. Your child should put it on when she/he wakes up in the morning until she/he goes to bed for sleeping at night. It is not waterproof and should be removed during bathing, showering and swimming.
Please remind your child to fill in the diary he/she received from us. In the diary he/she can write the exact time to put it off and on and the activities your child did during nonwear time.

Your child is supposed to wear the kids-meter from ………………………….. till …………………………… . Please remember her/him to bring the kids-meter back to school and give it to the teacher at ………………………..

If you or your child has *any* questions or concerns regarding the study, please do not hesitate to contact us.

Sincerely,

Dr Mai Chin A Paw
EMGO Institute, Vumc
Van der Boechorststraat 7
1081 BT Amsterdam
Phone:
Email:

INTRUCTIONS FOR KIDS-METER USE

- **WEAR** the monitor everyday ALL DAY for **the next 6 days**.

- **REMOVE** the monitor just before going to bed. Leave it on a table or dresser where you will be sure to see it and put it on first thing the next morning.

- **PUT** your monitor on each morning when you get out of bed.

- **WEAR** the kids-meter with the **STICKER ON TOP**.

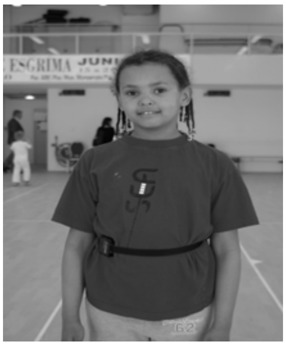

- **BE SURE** the monitor is on the right side of your waist. **NOT** at the front or back of your waist.

- **BE SURE** the monitor fits tightly around your waist. You can wear it under or above your clothes.

- **DON'T** drop the monitor.

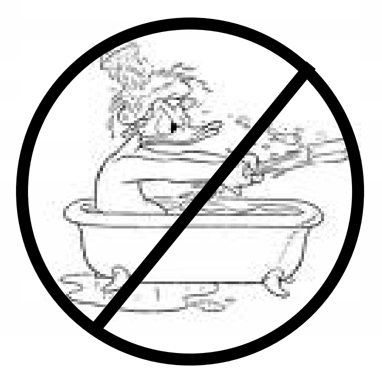

- **DON'T** let the monitor get wet. Remove the device during showering, swimming and bathing or any sports in which it can get wet.

- **BE SURE** to put it back on when you are out of the water.

- If you forget to put it on for any part of the day put it on as soon as you remember.

-**Please NOTE** at what time you put the accelerometer on and off and why in your diary.

- Please **DON'T** forget to bring it back to school after 6 days.
